# Author Correction to: Mixotrophic chemosynthesis in a deep-sea anemone from hydrothermal vents in the Pescadero Basin, Gulf of California

**DOI:** 10.1186/s12915-021-01067-4

**Published:** 2021-06-18

**Authors:** Shana K. Goffredi, Cambrie Motooka, David A. Fike, Luciana C. Gusmão, Ekin Tilic, Greg W. Rouse, Estefanía Rodríguez

**Affiliations:** 1 Occidental College, Los Angeles, LA USA; 2 Washington University, St Louis, MO USA; 3 American Museum of Natural History, New York City, NY USA; 4 University of Bonn, Bonn, Germany; 5 Scripps Institution of Oceanography, San Diego, CA USA

**Author Correction to: BMC Biol 19, 8 (2021)**

**https://doi.org/10.1186/s12915-020-00921-1**

Following publication of the original article [1], the authors would like to include missing permit information in the Acknowledgements section.

The following text needed to be added to Acknowledgements section:

We acknowledge, with gratitude, the granting of sample collection permits by la Dirección General de Ordenamiento Pesquero y Acuícola, Comisión Nacional de Acuacultura y Pesca (CONAPESCA: Permiso de Pesca de Fomento No. PPFE/DGOPA-200/18) and la Dirección General de Geografía y Medio Ambiente, Instituto Nacional de Estadística y Geografía (INEGI: Autorización EG0122018), with the associated Diplomatic Note number 18-2083 (CTC/07345/18) from la Secretaría de Relaciones Exteriores - Agencia Mexicana de Cooperación Internacional para el Desarrollo / Dirección General de Cooperación Técnica y Científica.

The original article [1] has been corrected.
