# Supplementary material for: Mixotrophic chemosynthesis in a deep-sea anemone from hydrothermal vents in the Pescadero Basin, Gulf of California
Source: BMC Biol. 2021 Jan 18;19:8. doi: 10.1186/s12915-020-00921-1 (PMC7812739; doi:10.1186/s12915-020-00921-1)
Supplement: Supplementary file 3 — Additional file 2: Table S2. 16S rRNA amplicon results. Number of total 16S rRNA amplicon reads, the Shannon Diversity index (H′) and the relative abundance (%) of the SUP05 group (based on 16S rRNA barcode amplification) associated with Ostiactis pearseae, water samples, and other Anthozoa from the Pescadero Basin vents. [file 12915_2020_921_MOESM2_ESM.docx]

**Supp Table 2**: Number of total 16S rRNA amplicon reads, the Shannon Diversity index (H’) and the relative abundance (%) of the SUP05 group (based on 16S rRNA barcode amplification) associated with *Ostiactis pearseae*, water samples, and other Anthozoa from the Pescadero Basin vents.

| ***Ostiactis pearseae*** | | | | |
| --- | --- | --- | --- | --- |
| **Auka vent field** | **Dive #-Sample #** | **# of reads** | **H′ index**^1^ | **% SUP05** |
| Matterhorn | S0193-R2 | 11596 | 2.13 | 96.4 |
| E. of Diane’s vent | S0193-A2 | 1852 | 2.79 | 70.9 |
| Z vent (top) | S0194-S2 | 2405 | 2.60 | 82.5 |
| S. of Z vent (small chimney) | S0200-R2w | 21299 | 2.67 | 87.0 |
| **JaichMaa ‘ja’ag vent field** |  |  |  |  |
| Abuelita | S0197 | 16566 | 2.90 | 64.4 |
| Abuelita | S0197-CM2 | 4356 | 2.75 | 86.2 |
| Abuelita | S0197-CM3 | 6028 | 2.97 | 70.9 |
| Weey ‘kual | S0199-S8 | 10220 | 2.61 | 69.4 |
| **Water Samples** | | | | |
| **Auka vent field** | **Dive #-Sample #** | **# of reads** | **H′ index**^2^ | **% SUP05** |
| E. of Diane’s vent | S0193-N2 | 15754 | 4.38 | 6.7 |
| **JaichMaa ‘ja’ag vent field** |  |  |  |  |
| Abuelita | S0197-N2 | 24802 | 4.91 | 6.0 |
| Weey ‘kual | S0199-N1 | 10457 | 4.77 | 5.5 |
| **Other Anthozoa** | | | | |
| **Auka vent field** | **Dive #-Sample #** | **# of reads** | **H′ index**^2^ | **% SUP05** |
| Matterhorn | S0193-S4 | 1377 | 2.87 | 0.0 |
| S. of Z vent (small chimney) | S0200-R2r | 2805 | 3.14 | 0.3 |
| E. of Diane’s vent | S0193-R3 | 40902 | 2.82 | 0.1 |
| Z vent (lower on structure) | S0194-R1 | 2858 | 3.64 | 0.4 |
| NW. of Z vent (diffuse flow) | S0194-R2 | 2759 | 3.19 | 0.3 |
| NW. of Z vent (diffuse flow) | S0194-S1 | 47970 | 2.46 | 0.0 |

^1^ based on OTUs, defined as 99% similar based on 16S rRNA
