# Supplementary figures and images for: Mixotrophic chemosynthesis in a deep-sea anemone from hydrothermal vents in the Pescadero Basin, Gulf of California
Source: BMC Biol. 2021 Jan 18;19:8. doi: 10.1186/s12915-020-00921-1 (PMC7812739; doi:10.1186/s12915-020-00921-1)

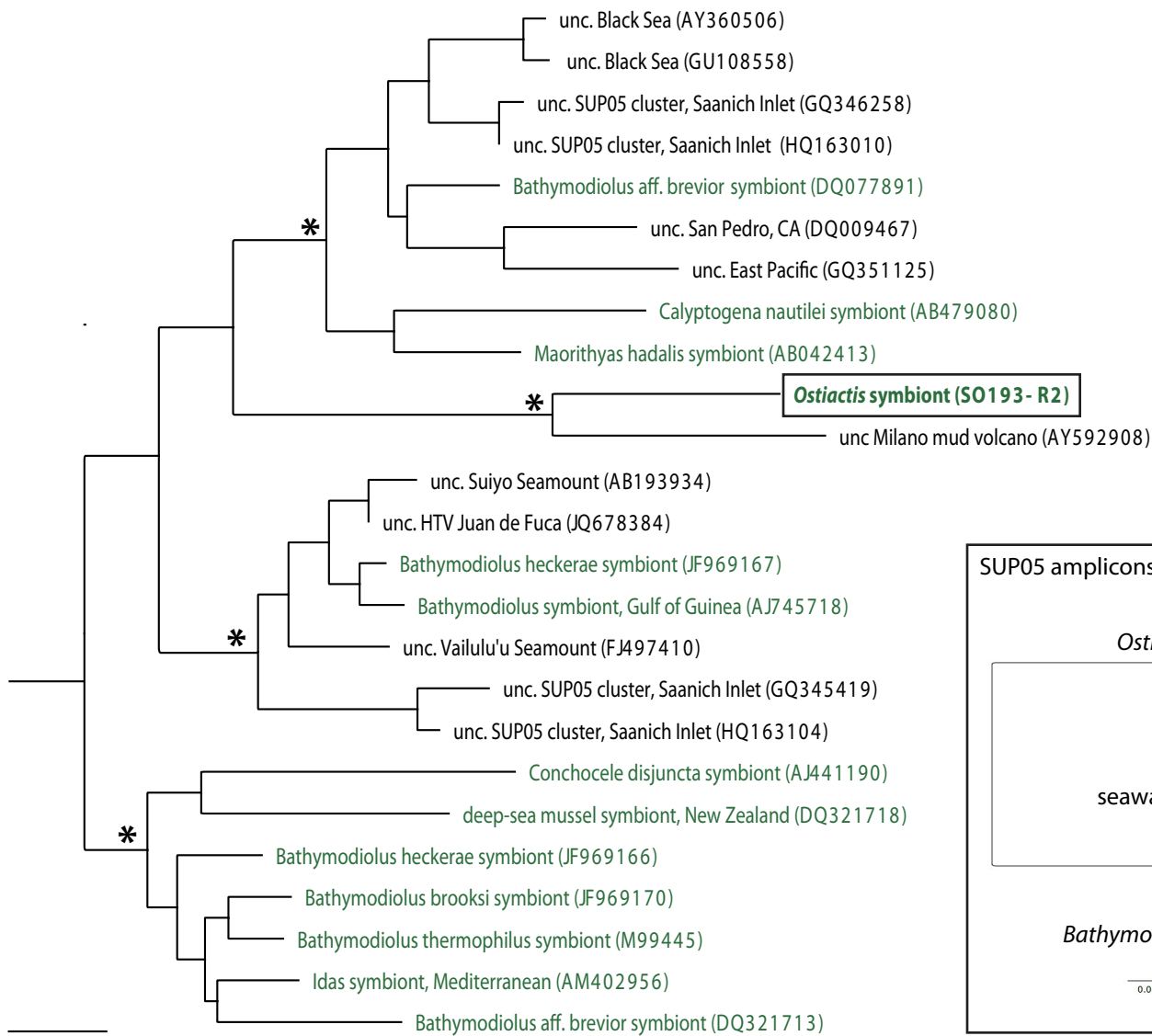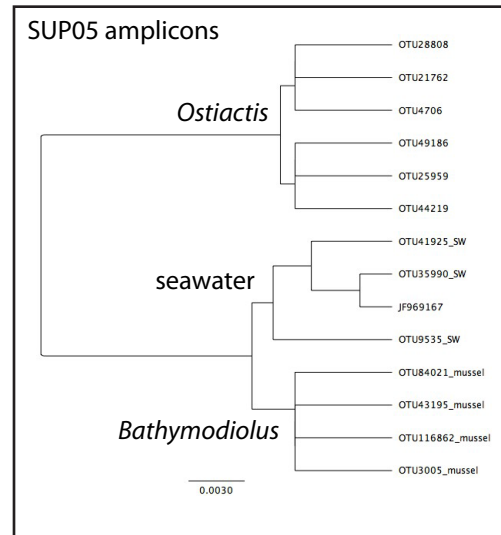

Supplement: Supplementary file 4 — Additional file 3: Figure S1. Phylogenetic relationships of the SUP05 group, based on 16S rRNA. A. SUP05 cluster, based on 16S rRNA. Taxa shown in green are known symbionts of marine invertebrates. * > 70% support (using the Jukes Kantor model). Additional taxa were included according to Petersen et al. 2012; Glaubitz et al. 2013; Shah et al. 2019. Inset. Shows SUP05 amplicons recovered from Ostiactis pearseae, surrounding seawater samples, and Bathymodiolus mussels from the Costa Rica margin Jaco Scar seep sites (SG, unpublished). [file 12915_2020_921_MOESM3_ESM.pdf]

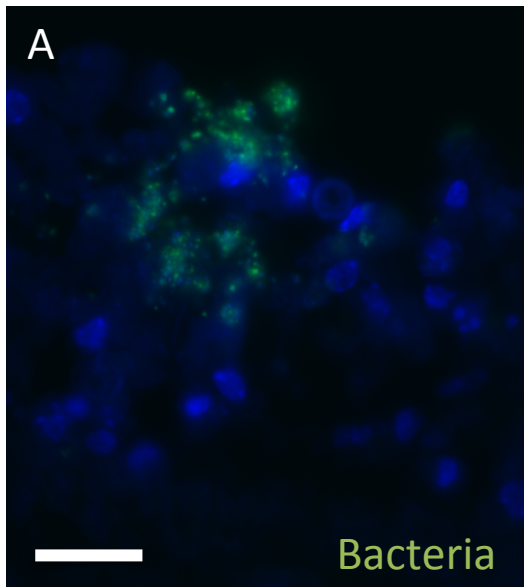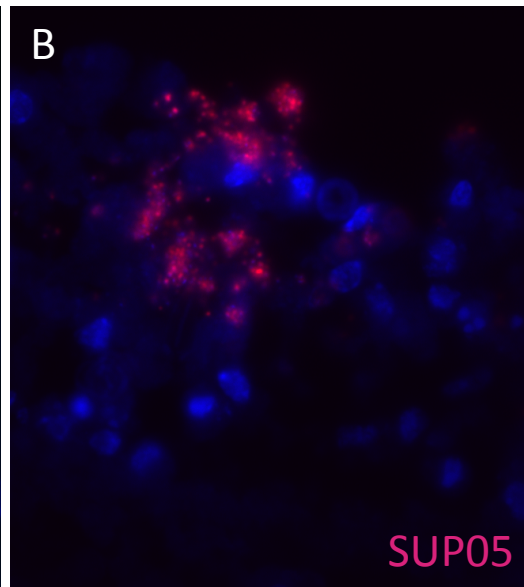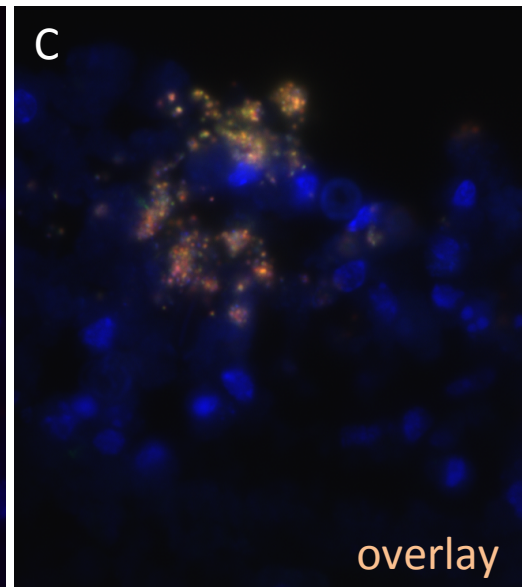

Supplement: Supplementary file 5 — Additional file 4: Figure S2. Fluorescence Microscopy of the tentacles of Ostiactis pearseae. Fluorescent in situ signal amplification via hybridization chain reaction-FISH (HCR-FISH) microscopy of Ostiactis pearseae tentacles using A. a general bacterial probe set Eub338 I-III, B. the specific Anem_SUP05 probe, and C. an overlay of the two showing near complete overlap. Scale is 10 μm. [file 12915_2020_921_MOESM4_ESM.pdf]
